# New Uterine Support for Horse-Women

**Published:** 1888-02

**Authors:** 


### NEW UTERINE SUPPORT FOR HORSEWOMEN.

R. J. Maitland Coffin, F. R. C. P. Ed., in the "British Medical Journal," says:

Shortly after my association with the "Wild West," I asked the frontier girls whether the violent riding did not shake them considerably, and was told that at times they suffered a great deal of pain. I advised them to get abdominal belts to keep themselves together whilst riding; they did so, and felt great benefit therefrom. On talking the matter over with Mr. E. Huxley, of Old Cavendish street, he suggested a uterine support, which consists of a peculiar shaped pad, with two straps which cross over the sacrum, and are brought to the front and fastened, with any amount of tightness the wearer may find desirable. I got him to make one for each of the girls, and they say they are such a comfort and support that they will never ride without them. I think the belt will prove a great blessing to ladies in the hunting field, as well as to professional riders.

